# Durability of GLP-1-Based Therapies in Obesity: Receptor Adaptation, Tachyphylaxis, and the Therapeutic Ceiling

**DOI:** 10.3390/jcm15218265

**Published:** 2026-10-23

**Authors:** Kylie Roslin, Robert Andrews, Brigitte Baella-Olivieri, Mitchell Roslin

**Affiliations:** 1 Miller School of Medicine, University of Miami, 1600 NW 10th Avenue, Miami, FL 33136, USA; krr65@miami.edu; 2 Barbara and Donald Zucker School of Medicine at Hofstra/Northwell, 500 Hofstra University, Hempstead, NY 11549, USA; randrews3@northwell.edu (R.A.); bbaellaolivieri@northwell.edu (B.B.-O.)

**Keywords:** GLP-1 analogues, weight loss, bariatric surgery, morbid obesity, tirzepatide, semaglutide

## Abstract

Glucagon-like peptide-1 (GLP-1) receptor agonists and related incretin-based therapies have transformed the treatment of type 2 diabetes and obesity. Their dependence on sustained receptor agonism, however, creates an unavoidable longitudinal question: what happens after 5–10 years when a patient has reached a stable plateau at the highest approved or tolerated dose? The answer cannot be inferred from short-term weight loss alone. At the therapeutic ceiling, stable benefit, partial attenuation of appetite or reward effects, progression of the underlying disease, increasing treatment burden, and renewed response to an additional mechanism are all plausible trajectories. This conceptual review argues that tachyphylaxis should be treated as a biologically plausible, testable component of long-term incretin pharmacology. Rapid tachyphylaxis to the gastric-emptying effect of GLP-1 has already been demonstrated. Because chronic weight regulation also depends on sustained signaling through neural and peripheral receptor pathways, partial attenuation of selected effects cannot be assumed absent simply because mean weight loss remains below baseline. Existing trials establish persistent activity for several years, but they were not designed to exclude effect-specific receptor adaptation during long-term stable-dose exposure. Weight-loss physiology also creates powerful competing explanations, including lower energy requirements, adaptive thermogenesis, neuroendocrine defense of body weight, behavioral accommodation, and dose constraints. Withdrawal studies establish treatment dependence, not the absence of on-treatment attenuation. Long-term durability should therefore be assessed across appetite and reward responses, weight maintenance, glycemic and cardiovascular benefit, beta-cell function, gastrointestinal burden, body composition, physical function, and residual disease. This framework is intended to help clinicians recognize a therapeutic ceiling, distinguish its possible mechanisms, and decide when to maintain, combine, switch, or add procedural therapy.

## 1. Introduction

GLP-1 receptor agonists were initially developed for the treatment of type 2 diabetes mellitus because of their glucose-dependent insulinotropic effects, suppression of glucagon secretion, and relatively low intrinsic risk of hypoglycemia. Weight loss emerged as an important secondary effect and subsequently became a primary therapeutic target. In pivotal obesity trials, once-weekly semaglutide 2.4 mg produced mean weight loss of 14.9% at 68 weeks, while tirzepatide produced mean reductions of 19.5% and 20.9% at the 10 mg and 15 mg doses, respectively [1,2].

An important distinction emerges when outcomes in obesity trials are compared with those in populations with type 2 diabetes. In STEP 2, semaglutide 2.4 mg produced mean weight loss of approximately 9.6% using the treatment-policy estimand, substantially less than the 14.9% observed in STEP 1 among participants without diabetes [1,3]. Similarly, tirzepatide trials in type 2 diabetes generally demonstrate less weight loss than obesity trials conducted in participants without diabetes [2,4]. The reasons are likely multifactorial and may include greater insulin resistance, concomitant medications, differences in beta-cell function, older age, and a metabolically more advanced disease phenotype. These observations support the hypothesis that metabolic phenotype influences responsiveness (Table 1).

Pivotal trials use gradual dose escalation primarily to improve gastrointestinal tolerability, but escalation also complicates interpretation of durability. Early rapid loss typically slows as treatment proceeds, and additional dose exposure may restore or extend response. That pattern can reflect planned titration, inadequate initial exposure, physiologic counter-regulation, or pharmacologic attenuation; current trial designs do not cleanly separate them. The practical dilemma becomes most evident when a patient reaches a high or maximal tolerated dose, has no remaining dose headroom, and still has substantial obesity or functional impairment.

Long-term evidence remains incomplete. SELECT showed that semaglutide-associated weight reduction and cardiovascular benefit can persist for approximately 4 years, clearly refuting complete loss of drug activity during continuous treatment [5]. Yet persistence of group-level benefit does not exclude partial attenuation of specific effects, heterogeneity among patients, or a waning response masked by continued benefit relative to baseline. Withdrawal studies show substantial regain after semaglutide or tirzepatide is stopped, confirming that ongoing exposure remains active and usually necessary [6,7,8]. The unresolved question begins where most trials end: after 5–10 years at a stable high or maximal dose, especially in patients with severe residual obesity, diabetes, or functional disease. Tirzepatide 15 mg and semaglutide 7.2 mg make this question increasingly relevant [2,9].

Trial efficacy also does not automatically translate into durable real-world effectiveness. Discontinuation, lower maintenance doses, shortages, insurance barriers, adverse effects, and less intensive follow-up all reduce achieved weight loss [10,11]. Durability must therefore be analyzed at two levels: the pharmacodynamic durability of receptor-based therapy under sustained exposure and the practical durability of a treatment model that depends on continuous access, adherence, and tolerability.

## 2. Representation of Advanced Obesity

Pivotal trials enrolled participants across a broad BMI range, but mean baseline BMI was generally in the high 30 s, and evidence is thinner for BMI values of 50, 60, or higher. Class IV obesity is rarely reported as a dedicated stratum. Average trial outcomes therefore cannot be assumed to define therapeutic adequacy in the most advanced disease.

The distinction is clinically consequential. A patient weighing 400 pounds who loses 20% remains at 320 pounds. The response is substantial, but severe mobility limitation, sleep-disordered breathing, heart-failure physiology, venous disease, and other mechanical or metabolic complications may persist. In advanced obesity, the relevant endpoint is not whether the drug worked, but whether the magnitude and durability of benefit are sufficient for the residual disease burden.

## 3. Tachyphylaxis: Definition and Clinical Relevance

Tachyphylaxis is a reduction in responsiveness after repeated or continuous drug exposure. Receptor phosphorylation, internalization or downregulation, altered intracellular signaling, neural adaptation, mediator depletion, and counter-regulatory pathways are established mechanisms. Although the term is often reserved for relatively rapid attenuation and tolerance for slower change, both describe a central pharmacologic issue: the same exposure produces less of a previously observed effect.

For a therapy built on chronic receptor agonism, tachyphylaxis is not a remote analogy; it is an expected pharmacologic possibility that must be tested effect by effect. GLP-1 biology already provides direct proof of principle. Continuous native GLP-1 exposure rapidly attenuates its slowing of gastric emptying, probably through vagal and receptor-linked pathways [12,13]. This does not establish identical adaptation in central appetite, reward, glycemic, or cardiovascular pathways, but it demonstrates that GLP-1-mediated effects are not uniformly protected from tachyphylaxis.

Sustained weight reduction over 2–4 years shows that long-acting therapy retains clinically important activity [5,14]. It does not resolve whether that activity is smaller than it was earlier, whether specific components such as satiety or food-reward suppression have attenuated, or whether dose escalation concealed a declining pharmacodynamic response. The appropriate hypothesis is therefore neither inevitable class-wide tolerance nor guaranteed durability, but effect-specific adaptation operating alongside physiologic counter-regulation.

Weight loss itself activates a powerful defense of body mass. Energy expenditure falls as body size declines and may decrease beyond that predicted by changes in fat and lean mass. Persistent changes in leptin, ghrelin, peptide YY, cholecystokinin, insulin, and other signals can increase hunger and reduce satiety [15,16,17]. These responses can oppose an otherwise active drug and produce a plateau without receptor desensitization.

A plateau is therefore a phenotype, not a mechanism. It may represent a new energy-balance equilibrium, partial receptor or neural adaptation, progressive disease, behavioral accommodation, inadequate exposure, or several of these together. The clinically informative signal is change at a stable dose: return of hunger, food preoccupation, cravings, or glycemic deterioration before substantial weight regain is more suggestive of pharmacodynamic attenuation than a stable plateau with preserved satiety and metabolic benefit.

Repeated escalation remains an important pharmacologic signal. Escalation may be appropriate and evidence based, but it should not be treated as physiologically neutral. When progressively greater receptor exposure is required to extend or recapture response, clinicians should ask whether marginal benefit remains proportional to adverse effects, cost, treatment burden, and loss of future therapeutic headroom.

## 4. Evidence for and Against Attenuation During Continued Treatment

### 4.1. Established Evidence

- Rapid tachyphylaxis has been demonstrated for GLP-1-induced slowing of gastric emptying during continuous exposure [12,13].
- Short-acting and long-acting GLP-1 receptor agonists differ in the persistence of their gastric-emptying effects, supporting effect-specific rather than class-wide interpretation [13,18].

### 4.2. Persistent Activity Does Not Exclude Partial Attenuation

- Semaglutide-associated weight reduction remained clinically meaningful during approximately 4 years of continued treatment in SELECT, demonstrating persistent activity but not measuring effect-specific pharmacodynamic change at a stable dose [5].
- In randomized withdrawal studies, continued-treatment groups maintained or deepened weight loss, while placebo-switch groups regained weight [7,8]. This proves ongoing drug activity and treatment dependence; it does not determine whether appetite, reward, or metabolic effects had partially attenuated before withdrawal.
- Energy-balance physiology predicts a plateau even when receptor signaling remains fully active. Conversely, a maintained mean weight below baseline cannot by itself exclude partial attenuation because the residual drug effect may still be substantial.

### 4.3. Unresolved Questions

The major unresolved question is whether prolonged stimulation produces partial adaptation in central appetite, reward, autonomic, or metabolic pathways. Dose escalation, slowing weight loss, and later return of hunger are compatible with that hypothesis but are not specific for it. The needed studies must hold dose and exposure stable while repeatedly measuring appetite, food intake, reward responses, energy expenditure, glycemia, and body-weight trajectory.

Central nervous system signaling deserves particular attention. GLP-1 receptors participate in distributed circuits governing satiety, food reward, autonomic function, and energy balance, and human neuroimaging demonstrates altered food-cue responses in the insula, amygdala, putamen, and orbitofrontal cortex during GLP-1 receptor activation [19]. Insulin acts in overlapping hypothalamic, striatal, and prefrontal networks, and reduced central insulin responsiveness has been described in obesity and type 2 diabetes [20]. Central neurotransmitter dysfunction is also part of DeFronzo’s expanded model of diabetes pathophysiology [21].

Leptin supplies a relevant precedent within weight regulation: obesity commonly combines high circulating leptin with an attenuated anorexigenic response. Proposed mechanisms include impaired blood–brain barrier transport, hypothalamic inflammation, endoplasmic reticulum stress, and intracellular negative feedback [22]. Central resistance to metabolic signals is therefore established as a biologic phenomenon. It strengthens the rationale for directly testing adaptation within GLP-1-responsive neural circuits, even though it does not by itself identify the responsible pathway.

Long-term response may reflect adaptation within GLP-1-responsive circuits, pre-existing insulin or leptin resistance, cross-talk among these systems, or counter-regulation caused by weight loss. These mechanisms are distinguishable only with longitudinal stable-dose studies combining quantitative appetite and food-reward measures, neuroimaging or other pharmacodynamic markers, and peripheral metabolic phenotyping. Until such studies are performed, partial central tachyphylaxis remains a credible unexcluded mechanism, not a conclusion that current evidence has disproved.

### 4.4. Beta-Cell Stress Versus Metabolic Unloading

A second receptor-level durability question concerns pancreatic beta-cell biology. The physiologic incretin effect accounts for approximately 50–70% of the insulin response to oral nutrients and reflects the combined actions of GIP and GLP-1 [23]. Pharmacologic incretin therapy amplifies this nutrient-responsive system at exposures well above endogenous hormone concentrations. Years of high-dose stimulation could increase secretory stress in patients with limited beta-cell reserve; at the same time, improved glycemia, weight loss, and reduced lipotoxicity may substantially unload the beta cell.

The sulfonylurea experience makes the concern clinically intuitive. In ADOPT, glyburide produced less durable glycemic control than metformin or rosiglitazone over approximately 5 years [24]. That finding does not establish secretagogue-induced beta-cell exhaustion because progressive diabetes, insulin resistance, glucotoxicity, and treatment history are inseparable. GLP-1-based therapy also differs fundamentally from sulfonylureas: insulin secretion is glucose-dependent and occurs alongside glucagon suppression, lower energy intake, weight loss, and improved insulin sensitivity.

We therefore propose a stress-versus-unloading model. Chronic incretin receptor stimulation increases secretory signaling, while lower glucose exposure, ectopic lipid flux, insulin resistance, and insulin demand reduce workload. The net effect may vary with beta-cell reserve and metabolic phenotype. Current evidence does not show accelerated beta-cell failure from GLP-1 receptor agonists, but available follow-up is not sufficient to close the question at 5–10 years of high-dose therapy. Extension studies should track insulin requirements, C-peptide-based measures when appropriate, and beta-cell function alongside weight and glycemia.

## 5. Dose Escalation, Gastrointestinal Adaptation, and Treatment Burden

GLP-1-based therapies influence gastric emptying, pyloric tone, intestinal signaling, nausea pathways, and central satiety. Nausea, vomiting, diarrhea, constipation, abdominal discomfort, and early satiety are dose-related and occur most often during escalation [1,2,3,4].

Higher-dose therapy makes this more consequential. In STEP UP, semaglutide 7.2 mg produced greater weight loss than 2.4 mg at 72 weeks, while gastrointestinal adverse events occurred in 70.8% and 61.2% of participants, respectively [9]. The study establishes incremental efficacy, not long-term tolerability. Stable-dose follow-up should measure persistent symptoms, retained gastric contents, clinically important dysmotility, nutritional compromise, and discontinuation.

Perioperative concern followed reports of retained gastric contents and aspiration. Broad withholding guidance issued in 2023 was replaced in 2024 by a more nuanced approach based on individual symptoms and characteristics [25,26].

Reports of ileus, pseudo-obstruction, intussusception, and bowel obstruction are being reported more frequently. Increased concerns have been raised following Roux-en-Y gastric bypass, SADI-S, and other operations that alter gastrointestinal anatomy. These events warrant prospective study.

Body composition is another component of treatment burden. Fat mass accounts for most of the loss in available semaglutide and tirzepatide substudies, but clinically meaningful lean-mass loss can occur [27,28]. Older adults, postmenopausal women, patients with sarcopenic obesity, and those with low protein intake or limited activity may be especially vulnerable.

Dose escalation should therefore be judged by marginal health benefit, not scale weight alone. Near the therapeutic ceiling, additional receptor exposure may yield less fat loss while increasing gastrointestinal symptoms, nutritional compromise, discontinuation, or lean-tissue loss. Long-term care should include adequate protein, resistance exercise, strength or functional assessment, and body composition when clinically useful.

## 6. Conceptual Comparisons with Other Drug Classes

Attenuation during chronic receptor-directed therapy is common enough across pharmacology to make long-term scrutiny of GLP-1 signaling obligatory. The following examples are not claims of mechanistic equivalence; they show how receptor adaptation, counter-regulation, and disease progression can produce similar clinical patterns and require different responses.

### 6.1. Opioids

Opioid tolerance involves receptor desensitization, altered signaling, and neural adaptation; escalation may restore analgesia while worsening treatment-related harm [29]. The relevant lesson is that the consequences of dose escalation were not obvious for many years. Certainly, GLP analogues are vastly different, but a major portion of their efficacy is based on central nervous system effects and binding to CNS receptors. A benefit has been reports of reduced addiction to alcohol and other addictive habits. Whether these benefits are sustainable or require dose escalation to maintain them is still uncertain.

### 6.2. Nitrates

Continuous nitrate exposure can produce tolerance within 24–48 h, and nitrate-free intervals may restore responsiveness [30]. This demonstrates how continuous stimulation can attenuate a therapeutic effect. GLP analogues seem to function differently, and long-term adherence seems mandatory but does not eliminate that there could be potential benefit from a cycled administration or from combination therapies at variable doses with a superimposed drug holiday.

### 6.3. Somatostatin Analogues

Somatostatin analogue doses are often increased over time to maintain the same therapeutic effect, suggesting tachyphylaxis. These gut hormones act through five G-protein-coupled somatostatin receptors that uncouple from the G protein. As a result, the same ligand concentration produces less intra-cellular signaling. The receptors on the surface are down-regulated. Additionally, there can physiologically counter regulation, as the body attempts to restore the secretions that somatostatin is trying to suppress. The relevance to GLP analogues is still uncertain. However, this demonstrates how a GI-produced hormone impact is reduced over time and requires dose escalation.

### 6.4. Beta-Agonists

Chronic beta-agonist exposure can reduce bronchoprotection through beta-2 receptor desensitization [31]. The clinical response is not indefinite escalation of one signal, but treatment of the underlying inflammatory disease with complementary mechanisms.

### 6.5. Interpretation

Together, these examples support a direct research agenda rather than a claim of equivalence. GLP-1 receptor agonists should be held to the same pharmacodynamic question asked of other chronic receptor-based therapies: at a stable exposure, does a defined biologic effect diminish over time, in whom, and by what mechanism?

## 7. Real-World Effectiveness and Durability

Randomized trials estimate efficacy under protocolized titration, uninterrupted supply, frequent contact, behavioral support, and selected eligibility criteria. Real-world durability also depends on insurance coverage, cost, availability, dose achieved, adverse effects, follow-up, preference, and persistence.

A 2025 Cleveland Clinic real-world cohort of nearly 8000 adults with obesity treated with injectable semaglutide or tirzepatide found substantially different outcomes according to persistence and maintenance dose. Patients who discontinued within 3 months lost approximately 3.6% at 1 year; those who discontinued between 3 and 12 months lost approximately 6.8%; and those who remained on therapy lost approximately 12%. Among persistent patients receiving higher maintenance doses, outcomes approached those observed in clinical trials [10].

These findings do not demonstrate intrinsic pharmacologic failure outside trials; they show that routine care often fails to reproduce trial exposure. The distinction is essential: interrupted access and submaximal dosing reduce effectiveness, whereas tachyphylaxis is a declining response despite sustained exposure. Both matter, but they require different solutions.

Discontinuation is commonly driven by cost, shortages, adverse effects, perceived lack of benefit, and preference [11]. Poorer real-world outcomes therefore cannot be assigned to receptor adaptation without exposure data. They do, however, reveal the fragility of a model that requires lifelong access, adherence, and tolerability.

### Dietary Quality and Long-Term Eating Behavior

GLP-1-based therapies reduce appetite, energy intake, cravings, and food preoccupation, creating an opportunity to improve eating behavior. Lower intake, however, is not synonymous with better dietary quality; marked appetite suppression and gastrointestinal symptoms can narrow food selection and compromise protein, fiber, micronutrients, and hydration [32,33].

Long-term nutrition should therefore be measured, not assumed. Adequate protein, dietary diversity, fiber-rich foods, hydration, and a cardioprotective pattern remain priorities. Whether healthier behaviors established during treatment persist after dose reduction or withdrawal is unknown and requires prospective study.

Medication should be integrated with resistance exercise, physical activity, behavioral support, and individualized dietary care. This is not an adjunctive courtesy: it determines whether weight loss improves body composition and function and whether benefit can be sustained when drug exposure changes.

## 8. The 5–10-Year Therapeutic Ceiling and the Limits of Pharmacologic Monotherapy

We define the therapeutic ceiling as the state in which a patient is receiving the highest approved, tolerated, or clinically acceptable exposure of a regimen, has reached a weight or metabolic equilibrium, and has no meaningful dose headroom. This is the point at which tachyphylaxis becomes clinically consequential even if it cannot yet be proven: further escalation is unavailable, and the adequacy of the remaining response must be judged against residual disease.

Five trajectories should be distinguished: durable maintenance with continuing health benefit; partial attenuation of appetite or reward effects; progression of metabolic or mechanical disease despite persistent drug activity; increasing gastrointestinal, nutritional, financial, or adherence burden; and renewed response when a different mechanism is added. A plateau alone cannot separate them, but longitudinal appetite, metabolic, functional, and exposure data can.

The objective is durable disease modification, not perpetual weight decline. A stable plateau with sustained cardiometabolic and functional improvement may represent success. The problem is a plateau with substantial residual disease, returning appetite, worsening glycemia, accumulating treatment burden, or loss of function. At that point, maintaining the same strategy simply because the drug remains partly active may be inadequate care.

### Can Newer Therapies Move the Ceiling?

The ceiling is likely to move as complementary pathways are added. In REDEFINE 1, cagrilintide plus semaglutide produced greater mean weight loss than semaglutide alone at 68 weeks [34]. Retatrutide, a GIP/GLP-1/glucagon receptor agonist, produced 24.2% mean weight reduction at 48 weeks at 12 mg, with the curve not clearly plateaued [35]. These studies expand short-term efficacy; they do not answer whether a higher ceiling remains durable.

Other agents may improve persistence rather than maximal efficacy. Oral nonpeptide GLP-1 agonists such as orforglipron may improve acceptability for some patients; in ATTAIN-1, orforglipron 36 mg produced 11.2% mean weight loss at 72 weeks [36]. Combinations targeting amylin, glucagon, GIP, or other pathways may alter efficacy and tolerability, but each will require the same stable-dose durability assessment. A newer drug may move the ceiling without eliminating receptor adaptation or the eventual plateau.

## 9. A Staged, Multimodal Model for Advanced Obesity

Advanced obesity should be managed according to disease burden and therapeutic adequacy rather than loyalty to a single modality. Nutritional, behavioral, pharmacologic, endoscopic, and surgical treatments can be deployed sequentially or concurrently when one mechanism does not produce sufficient magnitude, durability, or function. This approach does not require proof of tachyphylaxis; it requires recognition that partial drug activity may still be inadequate for advanced disease.

GLP-1-based therapies can serve as neoadjuvant agents to reduce liver volume, improve glycemia and mobility, and reduce perioperative risk before metabolic surgery. They can also be used after surgery for inadequate weight loss, recurrent weight gain, or persistent diabetes, and as bridging therapy during evaluation or optimization.

Metabolic and bariatric surgery provides the greatest average and most durable weight reduction for severe obesity. Guidelines support surgery at BMI 35 kg/m^2^ regardless of comorbidity and consideration at BMI 30–34.9 kg/m^2^ with metabolic disease or inadequate nonsurgical response [37]. Randomized and long-term studies demonstrate superior weight loss and glycemic control compared with medical therapy alone in appropriately selected patients [38,39]. Individual decisions still depend on disease burden, operative risk, expected benefit, prior response, access, and preference.

Medication and surgery should therefore not be framed as mutually exclusive competitors. The more useful question is which sequence or combination of mechanisms can deliver adequate and durable disease control with acceptable risk and burden.

## 10. Implications for Clinical Practice and Research

The long-term clinical question is not merely, “Has the patient stopped losing weight?” but, “Is the current receptor-based regimen still producing sufficient net health benefit for this disease burden?” Reassessment should determine whether appetite and reward control persist, glycemia and beta-cell function remain stable, gastrointestinal or nutritional burden is accumulating, body composition and function are preserved, and residual disease exceeds the capacity of the current regimen (Table 2).

The framework is longitudinal. The aim is to identify a change in trajectory at a known exposure: stable benefit, probable pharmacodynamic attenuation, disease progression, interrupted treatment, increasing burden, or insufficient magnitude. No single weight, symptom, or fasting insulin value can make that distinction.

- Obesity should be staged by disease burden, functional impairment, metabolic severity, and expected treatment response: not BMI alone.
- Trials should report outcomes separately for BMI ≥ 40, ≥50, and ≥60 kg/m^2^, with adequate representation of men, older adults, patients with diabetes, and patients with prior bariatric surgery.
- Long-term studies should distinguish stable-dose pharmacodynamic attenuation from adaptive energy-balance physiology, disease progression, dose escalation, nonadherence, and treatment interruption, and they should directly assess central GLP-1, insulin, and leptin signaling where feasible.
- Head-to-head studies should compare pharmacotherapy, surgery, endoscopic treatment, and combined strategies using weight, cardiometabolic events, functional status, body composition, quality of life, and cost.
- Perioperative and gastrointestinal studies should prospectively evaluate retained gastric contents, aspiration, ileus, obstruction, and outcomes in patients with altered gastrointestinal anatomy.
- Maintenance strategies should evaluate whether individualized nutrition counseling, dietary quality, physical activity, resistance exercise, protein optimization, lower-dose therapy, combination treatment, or procedural intervention can preserve health benefits while reducing adverse effects and cost.
- Public communication should distinguish ideal trial efficacy from real-world effectiveness and should avoid implying that average results apply uniformly to patients with extreme obesity.
- Dedicated extension studies should follow patients for at least 5–10 years, including prespecified cohorts maintained at stable high or maximal doses and separate analyses for classes III and IV obesity. Outcomes should include appetite and food reward, neural or other pharmacodynamic responses, energy intake and expenditure, weight and cardiometabolic benefit, beta-cell function, gastric emptying, gastrointestinal symptoms, body composition, persistence, and residual disease. This design is necessary to distinguish continued activity from partial tachyphylaxis and the ceiling of one drug from a ceiling moved by combination therapy.

## 11. Conclusions

GLP-1 receptor agonists and related incretin therapies are transformative treatments, but their long-term success depends on sustained receptor signaling. That fact makes tachyphylaxis a legitimate pharmacologic concern, not a criticism of the class. GLP-1-mediated slowing of gastric emptying already demonstrates effect-specific tachyphylaxis, establishing proof of principle that adaptation can occur within this system. The unanswered question is whether appetite, reward, autonomic, glycemic, or other effects also attenuate partially during years of stable high-dose exposure.

Current evidence shows persistent clinical activity for up to approximately 4 years; it does not exclude partial attenuation. A stable mean weight below baseline, a plateau, and regain after withdrawal answer different questions. None substitutes for longitudinal pharmacodynamic study at a stable dose. Receptor adaptation must also be separated from lower energy requirements, adaptive thermogenesis, neuroendocrine defense of body weight, behavioral accommodation, disease progression, treatment interruption, and inadequate therapeutic magnitude.

The therapeutic ceiling is the critical clinical decision point. Continued benefit may justify maintenance, but returning hunger, worsening glycemia, increasing gastrointestinal or nutritional burden, functional loss, or severe residual disease should prompt a change in strategy. Newer multi-receptor, amylin-based, and oral agents may raise the ceiling, while endoscopic and surgical therapies provide additional mechanisms. The appropriate response is not automatic escalation or automatic abandonment of pharmacotherapy, but deliberate selection of the combination that provides adequate and durable disease control.

## Figures and Tables

**Table 1:** Representative Weight-Loss Outcomes by Trial Population.

Trial or Program	Population	Baseline BMI	T2DM	Mean Weight Loss	Interpretation
STEP 1	Overweight or obesity	~37.9	No	14.9% at 68 weeks	High efficacy under structured trial conditions
STEP 2	Overweight or obesity and T2DM	~35.7	Yes	~9.6% at 68 weeks	Attenuated response in a diabetes population
SUSTAIN	T2DM	Commonly low 30 s	Yes	Generally ~4–7%	Lower-dose diabetes studies; not directly comparable
SURMOUNT-1	Obesity	~38.0	No	19.5–20.9% at 72 weeks	Highest pivotal monotherapy results at 10–15 mg
SUR	T2DM	Commonly low 30 s	Yes	Generally ~7–13%	Less weight loss than in obesity-only trials
SELECT	Overweight or obesity and CVD	~33.3	No	~10.2% at 4 years	Sustained long-term effect during treatment

Note: Cross-program comparisons are approximate because trial designs, estimands, treatment durations, doses, and populations differ. References [1,2,3,4,5].

**Table 2:** Primary-Care Reassessment at the Therapeutic Ceiling.

Domain	What to Assess	Key Interpretation	Potential Response
Persistent benefit	Weight and waist trajectory; HbA1c/glucose; blood pressure; lipids; sleep apnea, liver disease, mobility and other target comorbidities	A stable plateau with sustained metabolic or functional improvement can represent success	Continue effective dose; avoid escalation solely to force continued scale loss
Central response	Hunger, satiety, food preoccupation, cravings and reward-driven eating at a stable dose	Return of appetite before major weight regain is a clinically meaningful signal of possible attenuation; preserved satiety favors a stable equilibrium	Confirm adherence and exposures; consider behavioral contributors, adjunctive or alternative pharmacology, and further evaluation
Beta-cell/metabolic trajectory	HbA1c, fasting/postprandial glucose, diabetes medication and insulin requirements; C-peptide-based assessment when clinically indicated	Improved glycemia with lower insulin requirement supports metabolic unloading; worsening glycemia despite adherence may reflect disease progression or limited beta-cell reserve	Optimize diabetes therapy and assess residual metabolic disease rather than automatically attributing deterioration to the weight-loss drug
GI and nutritional burden	Nausea, vomiting, reflux, early satiety, constipation, hydration, protein intake, micronutrient risk and suspected dysmotility	GI burden may worsen, improve, or diverge from appetite efficacy	Adjust dose or regimen, investigate persistent symptoms, and protect nutrition and hydration
Body composition and function	Strength, resistance activity, protein intake, frailty risk and body composition when useful	Scale loss is not equivalent to favorable body-composition change	Prioritize resistance exercise and protein; reconsider intensity if functional loss outweighs incremental benefit
Residual disease and headroom	Current dose, tolerability, access, remaining obesity severity and mechanical/cardiometabolic complications	At maximal tolerated exposure, persistent advanced disease defines therapeutic inadequacy even if the drug remains active	Consider combination or newer agents, endoscopic therapy, metabolic surgery, or staged multimodal care according to risk and preference

## Data Availability

No new data were created or analyzed in this study. Data sharing is not applicable to this article.
